# Transparent, flexible graphene–ITO-based neural microelectrodes for simultaneous electrophysiology recording and calcium imaging of intracortical neural activity in freely moving mice

**DOI:** 10.1038/s41378-025-00873-y

**Published:** 2025-02-25

**Authors:** Miao Yuan, Fei Li, Feng Xue, Yang Wang, Baoqiang Li, Rongyu Tang, Yijun Wang, Guo-Qiang Bi, Weihua Pei

**Affiliations:** 1 Laboratory of Solid-State Optoelectronics Information Technology, Institute of Semiconductors, Chinese Academy of Sciences, Beijing, 100083 China; 2 Institute of Semiconductors, University of Chinese Academy of Sciences, Beijing, 10049 China; 3 Interdisciplinary Center for Brain Information, the Brain Cognition and Brain Disease Institute, Shenzhen Institutes of Advanced Technology, Chinese Academy of Sciences, Shenzhen, 518055 China; 4 Shenzhen-Hong Kong Institute of Brain Science, Shenzhen, 518055 China; 5 Hefei National Research Center for Physical Sciences at the Microscale, University of Science and Technology of China, Hefei, 230026 China; 6 School of Future Technology, University of Chinese Academy of Sciences, Beijing, 100049 China

**Keywords:** Electrical and electronic engineering, Biosensors

## Abstract

To understand the complex dynamics of neural activity in the brain across various temporal and spatial scales, it is crucial to record intracortical multimodal neural activity by combining electrophysiological recording and calcium imaging techniques. This poses significant constraints on the geometrical, mechanical, and optical properties of the electrodes. Here, transparent flexible graphene–ITO-based neural microelectrodes with small feature sizes are developed and validated for simultaneous electrophysiology recording and calcium imaging in the hippocampus of freely moving mice. A micro-etching technique and an oxygen plasma pre-treating method are introduced to facilitate large-area graphene transfer and establish stable low-impedance contacts between graphene and metals, leading to the batch production of high-quality microelectrodes with interconnect widths of 10 μm and recording sites diameters of 20 μm. These electrodes exhibit appropriate impedance and sufficient transparency in the field of view, enabling simultaneous recording of intracortical local field potentials and even action potentials along with calcium imaging in freely moving mice. Both types of electrophysiological signals are found to correlate with calcium activity. This proof-of-concept work demonstrates that transparent flexible graphene–ITO-based neural microelectrodes are promising tools for multimodal neuroscience research.

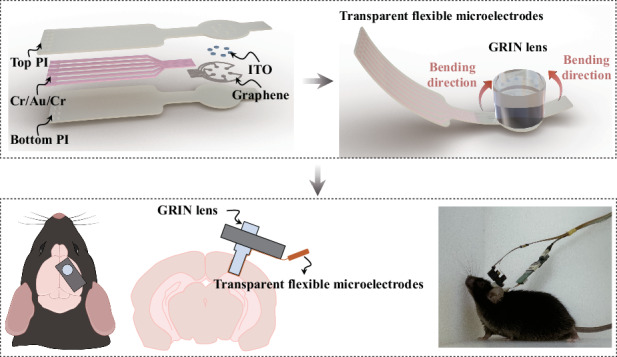

## Introduction

Neural activity recordings have significantly advanced understanding of brain functions, elucidating neurological disease mechanisms and developing effective treatments^1–3^. Electrophysiological techniques allow for high-temporal resolution recording of neural electrical activity and can detect signals across various spatial scales by optimizing electrode feature size, even for a single neuron^4–7^. However, these techniques face challenges in targeting specific cell types, resulting in a lack of spatial information. Conversely, calcium imaging provides cell-type specificity via genetically or chemically engineered calcium indicators. Nevertheless, the temporal resolution is constrained by the kinetics of calcium indicators binding to calcium ions^8^. Therefore, developing a unified tool integrating electrophysiology and calcium imaging would be invaluable, combining the strengths of each to achieve high spatiotemporal resolution in neural activity recording.

Conventional metal-based flexible neural electrodes are suboptimal for merging electrophysiology and calcium imaging due to the inherent opacity, which obstructs both light delivery and photon collection, thus impeding the field of view (FOV)^9,10^. Although metals designed with nano-network, nano-mesh, or nano-grid structures can achieve transparency^11,12^, serious electrophysiological signal distortion occurs when metals are exposed to light, triggering the photoelectric effect^13^. Indium tin oxide (ITO)^14^ is a commonly used material for transparent flexible neural microelectrodes (TFNMEs), offering good conductivity^14^, transparency^15^, and biocompatibility^16–18^. Nonetheless, its mechanical stability is compromised by the brittleness^19,20^. Other advanced transparent conductive materials, such as conductive hydrogels^21^, conductive polymers^22,23^, and carbon-based nanomaterials^24,25^ have been recently reported to overcome issues related to obscuration and light-induced artifacts. However, their inherent properties and fabrication methods typically result in recording sites of 50 μm or larger(several times the size of a neuron)^26^, substantially restricting the spatiotemporal resolution of neural activity recording. Consequently, miniaturizing the electrode’s feature size to that of a single neuron is essential for capturing high-frequency electrical activity during calcium imaging.

Graphene emerges as a promising candidate for TFNMEs owing to its transparency, flexibility, artifact-free, and biocompatibility^26–28^. Nonetheless, scaling down electrode feature size entails narrower interconnects and smaller recording sites, which typically increases electrode impedance, attenuates signal quality, and lowers defect tolerance. These challenges are exacerbated by difficulties in large-scale graphene transfer and instability of graphene-metal contacts, heightening the likelihood of open-circuit failures^29^. Ramezani et al.^30^ developed TFNMEs featuring 20 μm recording sites by interlayer-doping double-layer graphene (id-DLG) and electrochemically depositing platinum nanoparticles (PtNPs), though the electrochemical deposition method was unsuitable for batch production. Despite the small feature-size TFNMEs achieved simultaneous multiunit activity recording and two-photon calcium imaging, they were limited to a fixed mice cortical surface. Xue et al.^31^ proposed a tightly integrated neuronal imaging microscope (TINIscope) system that recorded local field potentials (LFPs) and calcium signals simultaneously in the hippocampus of mice using platinum-iridium electrodes assembled on the gradient refractive index (GRIN) lens. However, since the rigid electrodes were attached to the side of the lens, it remains uncertain whether the LFPs originated from the FOV, and the correlation between these potentials and the calcium signals has yet to be determined.

This work reports the development and validation of small feature-size TFNMEs based on graphene–ITO (Gr–ITO–TFNMEs) for high spatiotemporal resolution in intracortical neural activity recording. Gr–ITO–TFNMEs employ ITO as the interface material for recording sites, with metals serving as the interconnects and graphene acting as a bridge between the ITO and metal interconnects. The configuration ensures that the FOV is composed entirely of transparent materials and incorporates graphene flexibility, ITO biocompatibility, and superior conductivity, decreasing bending caused fatigue or damage, enhancing mechanical stability, and eliminating curling. A micro-etching technique is proposed to enable crack-free, wrinkle-free, and large-area graphene transfer through multiple alignments and transfers of small-area graphene. In addition, an oxygen plasma pre-treating the graphene method facilitates the stable, low-impedance contacts between graphene and metals. These technological advancements promote the batch production and high-quality fabrication of Gr–ITO–TFNMEs with small feature sizes (interconnect widths of 10 μm and recording site diameters of 20 μm). The resulting Gr–ITO–TFNMEs exhibit an electrochemical impedance of 1.45 MΩ at 1 kHz and demonstrate an optical transmittance greater than 80% at 520 nm. Finally, the ‘watch-style’ Gr–ITO–TFNMEs are integrated onto the GRIN lens of the TINIscope system, simultaneously recording electrophysiological signals and performing calcium imaging in the hippocampus of freely moving mice. The integrated Gr–ITO–TFNMEs can stably and reliably record LFPs and action potentials. The power spectral density of LFPs and the number of action potentials showed correlations with calcium activity.

## Results

### Design of the Gr–ITO–TFNMEs

**Fig. 1:**
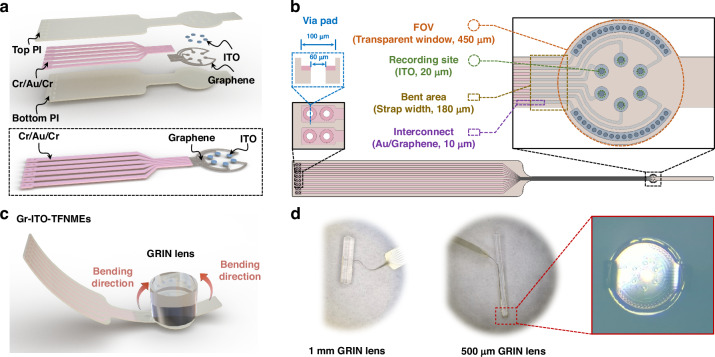
Design and integration of Gr–ITO–TFNMEs with the GRIN lens. **a** Layout of Gr–ITO–TFNMEs, with the dashed box illustrating the bridging configuration between ITO, graphene, and metal interconnects (Cr/Au/Cr). **b** Design of Gr–ITO–TFNMEs. **c** Schematic diagram of Gr–ITO–TFNMEs integrated with the GRIN lens. **d** Actual photographs of Gr–ITO–TFNMEs integrated with the GRIN lenses of varying sizes (1 mm and 500 μm in diameter)

Given the poor interfacial behavior of graphene-based TFNMEs (Gr-TFNMEs) when scaling down electrode feature size to single-cell size and the fundamental requirement for transparency and flexibility of TFNMEs, several material adaptations were made: ITO was utilized as the interface material for the electrodes, replacing graphene; metal interconnects (Cr/Au/Cr) were employed in the backend to reduce the trace impedance; and graphene was solely used as a conductive bridge between ITO and Cr/Au/Cr. The bridging method overcomes the brittleness of ITO and optimizes conductivity without compromising transparency and flexibility. The detailed configuration is depicted in the dashed box in Fig. 1a. The introduction of ITO allowed for Polyimide (PI) 2600 series (DuPont, HD Microsystems) to serve as the bottom and top insulation layers of the electrodes, compatible with dry etching and offering excellent tensile strength and transparency (compared to other commonly used insulation materials, as shown in Table S1). The layout of Gr–ITO–TFNMEs is illustrated in Fig. 1a. To stably integrate Gr–ITO–TFNMEs with the objective lens (GRIN lens) of the TINIscope system, the electrodes were designed like a ‘watch-style’ structure, as shown in Fig. 1b. The ‘watch dial’ represents the FOV and was aligned with the end face of the lens, serving as the transparent window for calcium imaging. Six recording sites, each with a diameter of 20 μm, were centrally distributed on the FOV to record neural electrical activity. The ‘watch strap’, with a width of 180 μm, was designed to bend at 90°and attach to the side of the lens to reinforce the integration. The bent area contained no ITO; a PI–graphene/Au–PI configuration was employed. The graphene/Au traces were designed with a width of 10 μm. The method of bending Gr–ITO–TFNMEs to integrate with the lens is illustrated in Fig. 1c. The high tensile strength of PI (350 MPa) and graphene (10–50 GPa) enable them to be seamlessly and non-destructively integrated into GRIN lens with diameters of 1 mm or even 500 μm, as displayed in Fig. 1d.

### Fabrication of the Gr–ITO–TFNMEs

**Fig. 2:**
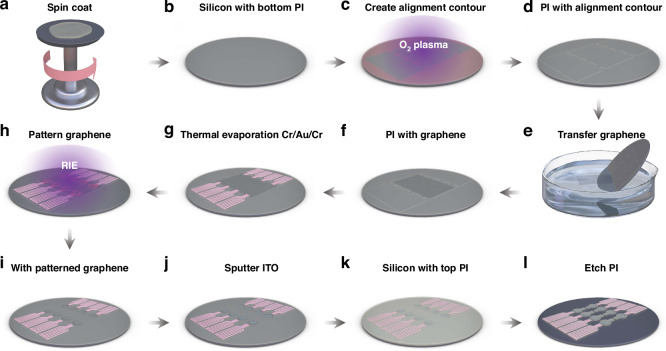
Fabrication of Gr–ITO–TFNMEs. **a** Spin-coating PI on the silicon wafer. **b** Silicon wafer with bottom PI. **c** Creating alignment contours on the bottom PI by oxygen plasma treatment under a photoresist mask. **d** Bottom PI with alignment contours. **e** Transferring graphene based on the alignment contours. **f** Bottom PI with graphene. **g** Thermal evaporation deposition of Cr/Au/Cr after oxygen plasma pre-treating. **h** Patterning graphene by RIE under a photoresist mask. **i** Patterned graphene. **j** Sputtering ITO. **k** Spin-coating the top PI. **l** RIE PI to define the outline of Gr–ITO–TFNMEs, connection pads and openings of recording sites

During the fabrication of TFNMEs containing graphene, two major challenges were encountered: (1) The surface tension of water complicated large-area graphene transfer. It is difficult to spread the large-area graphene out smoothly on the substrate without overlaps or wrinkles; (2) The hydrophobic nature of the graphene surface hindered the formation of stable, low-impedance contacts with metals. We proposed a micro-etching technique and pre-treating the graphene with oxygen plasma to address these challenges and achieve the batch production and high-quality fabrication of Gr–ITO–TFNMEs. The micro-etching technique utilized a photoresist mask for oxygen plasma treatment to create alignment contours on the PI, enabling large-area, cracks-free, and wrinkle-free graphene transfer through multiple alignments and transfers of small-area graphene. Pre-treating the graphene with oxygen plasma hydrophilized the surface, allowing metals to be uniformly deposited and form low-impedance contacts. These treatments significantly enhanced the yield of electrode fabrication. The fabrication of Gr–ITO–TFNMEs contained the following 9 steps, as shown in Fig. 2. (1) PI was spin-coated and cured to form the 3 μm bottom insulation layer on the 4-inch silicon wafer. (2) Alignment contours (~100 nm) were formed on the bottom PI by oxygen plasma treatment under a positive photoresist (MICROPOST S1805 G2) mask. (3) Suspended self-help transfer few-layer graphene (Xianfeng Nano, 800–1000 Ω/▫) was aligned face-to-face with the alignment contours and sequentially transferred to the target areas on the surface of deionized water. After baking at 150 °C to reduce wrinkles, as demonstrated in Fig. S1, the graphene was soaked in acetone to remove polymethyl methacrylate (PMMA). (4) Cr/Au/Cr with a thickness of 10/100/10 nm were thermally evaporated to form metal interconnects through a lift-off process after being treated by oxygen plasma. As shown in Fig. S2, this treatment facilitated uniform and stable deposition of metals. Additionally, low-impedance contacts were created between graphene and metals, validated by measuring the impedance of short-circuited metal interconnects to graphene, found to be approximately 1 kΩ. (5) The graphene was patterned by reactive ion etching (RIE) with a photoresist mask (AZ-6130). (6) A 140 nm of ITO was magnetron sputtered to form the interface of recording sites with a lift-off process. (7) A 3 μm layer of PI was spin-coated and cured as the top insulation layer. (8) The outline of Gr–ITO–TFNMEs, connection pads, and openings of recording sites were defined by RIE with a photoresist mask (AZ-4620). (9) The entire silicon wafer was submerged in deionized water to release the electrodes. The detailed fabrication processes are described in the Materials and methods. The morphology of the recording sites was characterized by SEM, as shown in Fig. S3. The SEM results indicate that the surface morphology of the electrode is uniform.

The entire process significantly enhanced the electrode quality for batch fabrication through a micro-etching technique and oxygen plasma pre-treating, achieving the preparation of Gr–ITO–TFNMEs with small feature size. The similar fabrication processes of Gr–TFNMEs and ITO-based TFNMEs (ITO–TFNMEs) are described in Supplementary Information (Notes S1 and S2). For easy plug-and-play in vitro validation and animal experiments, a custom flexible printed circuit board (FPC) was designed to connect to test equipment such as electrochemical workstations or amplifiers for electrophysiology recording. The Gr–ITO–TFNMEs and the FPC were connected via gold ball bonding^32,33^, replacing the commonly used anisotropic conductive film (ACF) thermal bonding^34^. Under ultrasonic pressure, the gold balls were electrically connected through circular vias of the square welding pads on the Gr–ITO–TFNMEs to the pads on the FPC, followed by encapsulation and reinforcement with sealing glue, as depicted in Fig. S4.

### Integration of Gr–ITO–TFNMEs with the GRIN lens

**Fig. 3:**
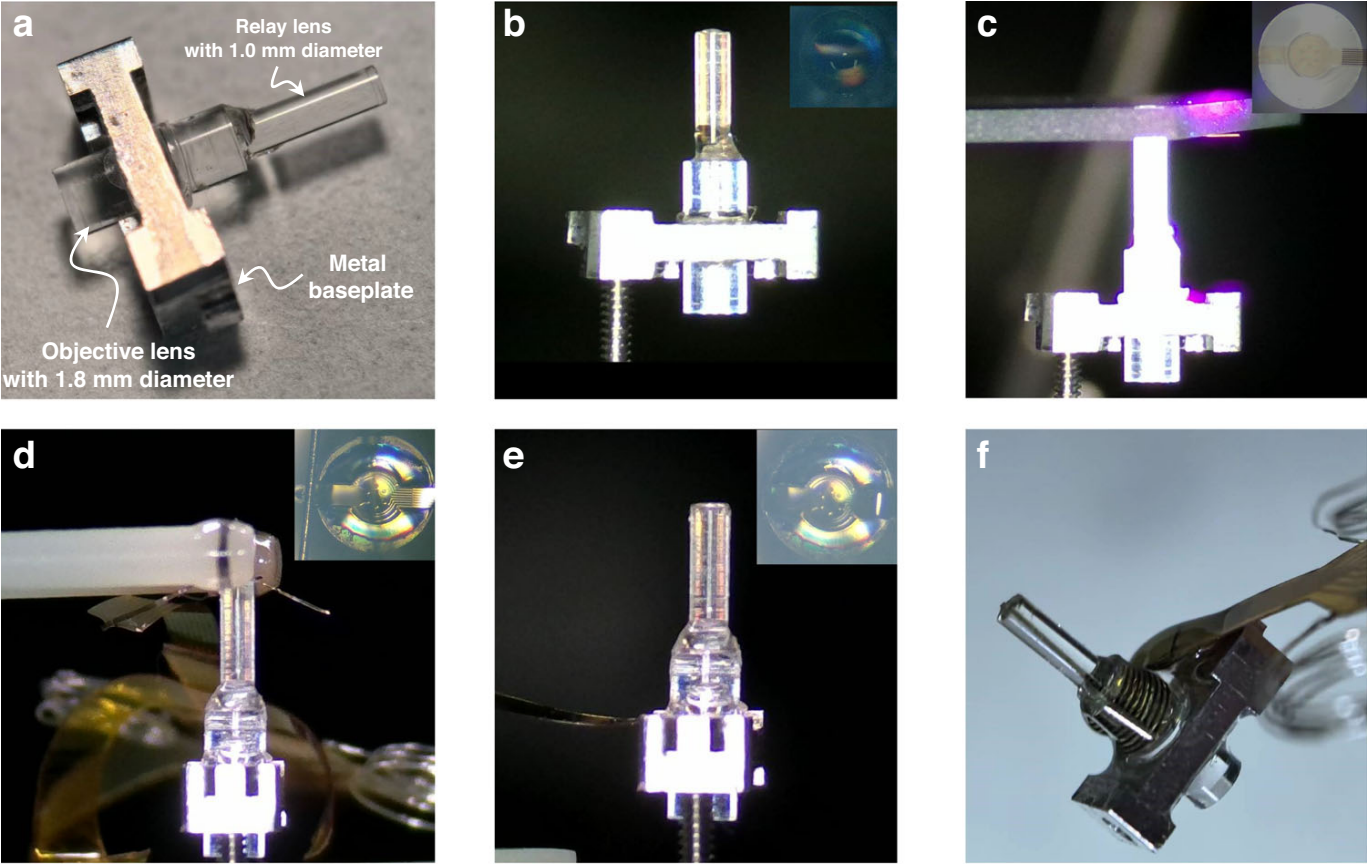
Integration of Gr–ITO–TFNMEs and the GRIN lens. (Insets show the top view of the lens). **a** Actual photograph of the integrated 1.8 mm diameter objective lens, 1.0 mm diameter GRIN relay lens, and metal baseplate. **b** The lens after leveling and applying optical adhesive. **c** Alignment and fixation of the electrode ‘dial’ and lens; **d** Fixation of the electrode ‘strap’ using a wire tool. **e** Fixation of the electrode ‘strap’ completed. **f** Actual image of the integrated electrode and lens

The TINIscope system was a miniaturized integrated fluorescence microscope for brain imaging^31^. The excitation light was focused through a series of optical components and an objective lens onto the neurons. The resulting calcium fluorescence was collected by the same lens, routed through additional optical components, and finally focused onto an image sensor. The integration of Gr–ITO–TFNMEs with the TINIscope system involved aligning and adhering the end face of the objective lens to the FOV of electrodes, ensuring stable connectivity to the electrophysiological recording system without disrupting optical functionality. To minimize tissue damage during lens implantation, a 1 mm GRIN relay lens was affixed to the objective lens using optical adhesive, as shown in Fig. 3a. The objective lens was bonded to a customized metal baseplate with two M1 threads to secure the microscope and level the lens. The detailed integration of Gr–ITO–TFNMEs with the GRIN lens was conducted using micromanipulator operations and followed five steps, as depicted in Fig. 3b–f: (1) Fixing and leveling the lens using a lens holder; (2) Applying optical adhesive to the end face of the lens using a fine-gauge needle; (3) Attaching the electrode in reverse to a glass slide, aligning the electrode ‘dial’ with the end face of the lens using a slide holder, and curing optical adhesive; (4) Using a wire tool to attach the electrode ‘strap’ vertically downward from the cut face of the lens; (5) Winding the unattached part of the electrode around the 1.8 mm diameter GRIN lens and securing the electrode and FPC to the baseplate with flexible silicone. The detailed structures of the customized lens holder, slide holder, and wire tool are provided in Fig. S5.

### Electrochemical characterization

**Fig. 4:**
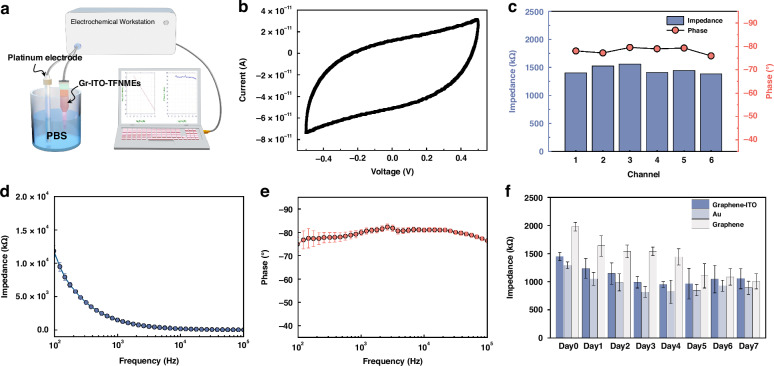
Electrochemical performance of Gr–ITO–TFNMEs. **a** Schematic diagram of the setup for electrochemical characterization. **b** CV characteristics of a representative Gr–ITO–TFNME. **c** Electrochemical impedance distribution at 1 kHz across six recording sites on a representative Gr–ITO–TFNME. **d**, **e** Averaged EIS across six recording sites on a representative Gr–ITO–TFNME (*n* = 6). **f** Accelerated aging test results of the three different interface electrodes for one week (with the impedance averaged across six recording sites for each representative electrode (*n* = 6))

The ability of electrodes to record electrophysiological signals is typically evaluated using electrochemical performance. The electrochemical characterization of Gr–ITO–TFNMEs was conducted in 0.1 M phosphate-buffered saline (PBS) with the Gr–ITO–TFNMEs serving as the working electrode, with a platinum electrode functioning as both the counter and reference electrode, as shown in Fig. 4a. Figure 4b presents the cyclic voltammetry (CV) characteristics as a representative sample, which displays a complete envelope without redox peaks. The result indicates a uniform electrode surface with capacitive characteristics consistent with the SEM results, which means the desired interface properties of neural microelectrodes. Figure 4c–e shows the electrochemical impedance at 1 kHz distribution across 6 recording sites and the averaged electrochemical impedance spectroscopy (EIS) over a frequency range from 100 Hz to 100 kHz. The improved fabrication process results in an acceptable electrochemical impedance, approximately 1.45 MΩ, within the range acceptable for single-unit recording. The impedance is slightly higher than that of same-sized ITO–TFNMEs (as shown in Fig. S6), attributed to the high sheet resistance caused by the two-dimensional atomic structure and grain boundaries of graphene. To evaluate the capability for sustained recording, an accelerated aging test was carried out by soaking the electrodes in PBS at 67 °C for one week, comparing three different types of electrodes: Gr–ITO–TFNMEs, Gr–TFNMEs, Au-based flexible microelectrodes (Au-FNMEs, as control). As shown in Fig. 4f, the Gr–ITO–TFNMEs exhibit the same impedance change trend as the Au-FNMEs, indicating comparable stability. In contrast, the Gr-TFNMEs show a continuous decrease in impedance, which is suspected to result from delamination between the top insulating layer and the graphene.

### Optical characterization

#### Transmittance measurements

**Fig. 5:**
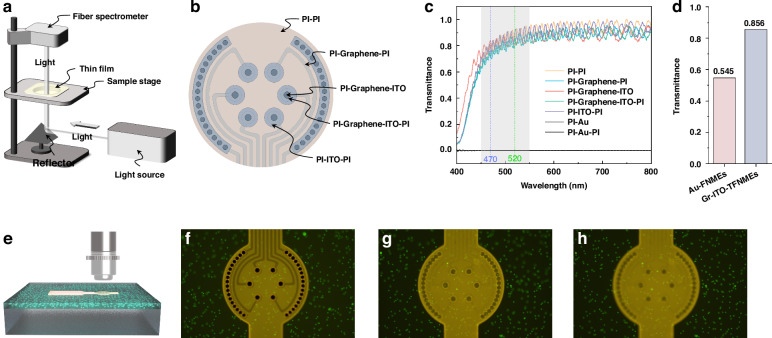
Optical characterization. **a** Schematic diagram of the transmittance measurement system. **b** Schematic diagram of different material combinations in the FOV. **c** Transmittance results for a representative sample. **d** Weighted average transmittance across area formed by different material combinations for Au-FNMEs and Gr–ITO–TFNMEs based on (**c**). **e** Schematic diagram of imaging fluorescent polystyrene microbeads. **f** Imaging focused on the surface of Au-FNMEs. **g** Imaging focused on the surface of Gr–ITO–TFNMEs. **h** Imaging focused below the surface of Gr–ITO–TFNMEs

The transmittance of Gr–ITO–TFNMEs was measured using a fiber optic spectrometer, as described in our previous work^10^. The schematic diagram of the setup is shown in Fig. 5a. To characterize the impact of different materials on transmittance in the FOV, various combinations were measured: PI–PI, PI–Graphene–PI, PI–Graphene–ITO, PI–Graphene–ITO–PI, and PI–ITO–PI, corresponding to different imaging regions as shown in Fig. 5b. Additionally, to compare with traditional metal electrodes, the transmittance of PI–Au–PI and PI–Au combinations was also measured. The optical transmittance of different regions of Gr–ITO–TFNMEs and Au-FNMEs is shown in Fig. 5c. The Au-FNMEs completely obstructed light propagation due to the inherent opacity of metal. There are notable differences in transmittance among recording sites (PI–Graphene–ITO, PI–ITO–PI, PI–Graphene–ITO–PI), interconnects (PI–Graphene–PI), and other non-functional areas of FOV (PI–PI). The overall light transmittance at the wavelength of 520 nm is above 80%. These differences in transmittance across different regions can aid in specifically marking electrodes during calcium imaging. The weighted average transmittance across areas of different material combinations for Au-FNMEs and Gr–ITO–TFNMEs is shown in Fig. 5d, where the overall transparency of Gr–ITO–TFNMEs is significantly improved compared to that of Au-FNMEs. Additionally, an oscillation in transmittance throughout the entire FOV was observed, attributed to thin-film interference, consistent with previous work^10^.

#### Imaging of fluorescent polystyrene microbeads

To characterize the impact of Gr–ITO–TFNMEs on calcium imaging, 5 μm diameter fluorescent polystyrene microbeads were utilized to simulate neurons. Au-FNMEs and Gr–ITO–TFNMEs were positioned on polydimethylsiloxane (PDMS, Sylgard 184, Dow Corning) containing a low concentration of fluorescent microbeads to compare their effects on imaging, as shown in Fig. 5e. The detailed fabrication process for the PDMS containing a low concentration of fluorescent microbeads is provided in Materials and methods below. Figure 5f–h shows the imaging of 5 μm diameter fluorescent polystyrene microbeads through Au-FNMEs and Gr–ITO–TFNMEs, including areas within and outside the FOV. The Au-FNMEs fully block the imaging of microbeads at recording sites and interconnects, significantly impairing imaging. In contrast, imaging focused on the surface (Fig. 5g) and below the surface (Fig. 5h) of Gr–ITO–TFNMEs clearly shows the microbeads, indicating that the electrodes have little effect on fluorescence imaging. Higher magnification imaging is presented in Fig. S7a distinctly shows the fluorescent microbeads under recording sites and interconnects, while Fig. S7b shows areas both within and outside the FOV of Gr–ITO–TFNMEs, demonstrating that although the light intensity of the microbeads was still relatively weakened, the fluorescent microbeads can be imaged clearly through recording sites.

### Electrophysiology recording and calcium imaging in vivo

**Fig. 6:**
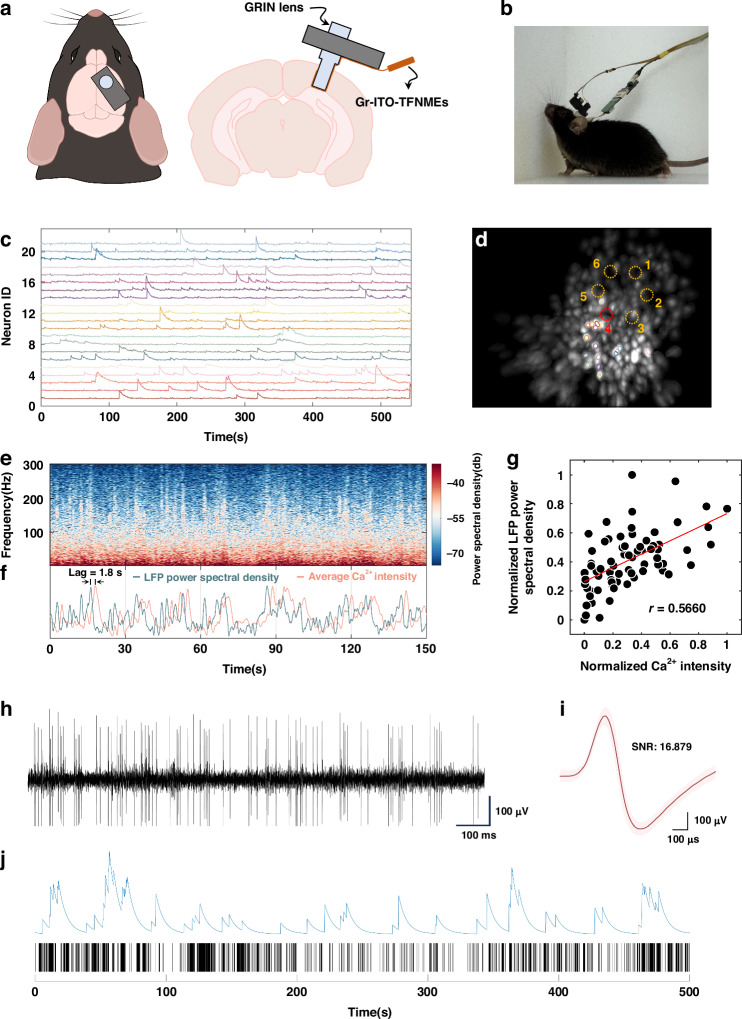
Simultaneous electrophysiology recording and calcium imaging. **a** Schematic diagram of the implantation of a GRIN lens integrated with Gr–ITO–TFNMEs. **b** Actual image of a freely moving mouse equipped with the TINIscope system and the electrophysiological recording circuitry. **c** Calcium traces of example neurons. **d** Spatial contours of example neurons in (**c**) and the corresponding maximum intensity map (MIP) of the background-subtracted videos, six recording sites are indicated by dashed circles. **e** Representative power spectral density of the LFP for recording site 4 in (**d**). **f** Representative average calcium intensity and the LFP power spectral density change over time, both normalized. **g** Scatter plot of the average calcium intensity and the LFP power spectral density with a correlation of 0.5660. **h** Neural signal acquired from Gr–ITO–TFNMEs after 300 Hz high-pass filtering. **i** The waveform after spike sorting. The waveform was derived from 4506 spikes recorded from a single neuron during the recording process. The light pink shaded area represents the variance, indicating spike-to-spike variability. **j** Representative calcium trace (top) and the spike raster plot (bottom) with the maximum correlation

To validate the ability of simultaneous electrophysiology recording and calcium imaging, the GRIN lens integrated with Gr–ITO–TFNMEs was implanted to the surface of the CA1. Male C57BL/6J mice (8 weeks) were anesthetized and fixed on a stereotaxic apparatus. A total of 400 nl of AAV2/9-hSyn-GCaMP7s virus was injected into the dorsal hippocampus (AP: −2.1 mm, ML: +1.7 mm, DV: −1.4 mm from skull surface with a 9° angle) at a rate of 15 nl/min. Three weeks later, the GRIN lens integrated with Gr–ITO–TFNMEs was implanted to the surface of the CA1 (DV: −1.15 mm below the surface of the skull) after the cortex above the CA1 was removed and secured to the skull using dental cement, as shown in Fig. 6a, b. After four weeks of recovery and adaptation, simultaneous electrophysiology and calcium imaging were conducted in freely moving mice. The detailed operation processes are described in Fig. S8 and “Materials and methods”.

Figure S9 shows the expression of GCaMP7s in the hippocampus. After integrating Gr–ITO–TFNMEs, the TINIscope system maintains high-quality identification of individual neurons (Fig. 6c, d and Supplementary Video S1), confirming that the introduction of Gr–ITO–TFNMEs has minimal impact on calcium imaging. Importantly, no light-induced artifacts were observed when the excitation light was turned on (Fig. S10). The representative power spectral density of the LFP for recording site 4 is demonstrated in Fig. 6e. The average calcium intensity across the FOV is changed following the LFP power spectral density with a delay of approximately 1.8 s, as shown in Fig. 6f. The recalibrated plot of the average calcium intensity and the LFP power spectral density is shown in Fig. S11d after shifting the average calcium intensity forward by 1.8 s. Such delays are commonly observed in experiments involving simultaneous electrophysiology recording and calcium imaging. Previous studies^35–37^ have reported delays in calcium signals relative to LFPs ranging from tens of milliseconds to seconds. Variations in delays may be attributed to differences in experimental conditions (such as FOV, imaging rate, and probe type) and differences in tissue structure and species. After accounting for the time delay, the correlation between the average calcium intensity and the LFP power spectral density over the entire recording duration was calculated using peak detection (Materials and methods). The resulting correlation coefficient reaches 0.5660, as shown in Fig. 6g. The high correlation between the calcium intensity and the LFP power spectral density within the FOV suggests that the calcium signal and electrophysiological signals originate from the same population of neurons. The power spectral densities of the LFPs from the other five recording sites and their correlations with the average calcium intensity are presented in Fig. S12.

Simultaneously, the Gr–ITO–TFNMEs recorded single neuron spikes in the hippocampus. It was the first instance of simultaneous single neuron activity recording and calcium imaging intracortically with TFNMEs, as shown in Fig. 6h, i. The signal-to-noise ratio (SNR) of the spikes reached up to 16, exemplifying the exceptional capability of Gr–ITO–TFNMEs in recording electrophysiological signals. The auto-correlogram of the detected neuron (Fig. S13a) displayed clear symmetry and a rapid decrease to baseline at the central peak, and the interspike interval histogram (Fig. S13b) showed an exponential decay, indicating a single neuron. Although changes in calcium signal intensity do not directly correspond to changes in action potentials, there is a significant correlation between calcium peaks and the number of spikes within the calcium peak duration^8^. Figure 6j shows the representative calcium trace (top) and the spike raster plot (bottom) that exhibit maximum correlation (*r* = 0.7846), which was obtained by traversing all neurons’ calcium peaks and the corresponding number of spikes. Fluctuations in the calcium trace are generally associated with intense spike activity occurring within brief periods; However, not all instances of intense spike activity lead to high peaks in the calcium trace. This discrepancy is speculated to be related to the location of calcium proteins, with spikes possibly occurring in regions lacking calcium proteins.

## Discussion

We developed a powerful tool for multimodal neural activity recording and addressed the challenges in the application of TFNMEs from three main perspectives.

First, in electrode design: Graphene and ITO, as commonly used transparent conductive materials^38^, are frequently combined in transparent electrodes for applications such as photodetectors and electrochemical detection, as shown in Table 1. This study is the first to combine graphene and ITO for multimodal neural activity recording. Here, graphene serves as a conductive bridge between ITO and the metal interconnects, overcoming the brittleness of ITO and optimizing conductivity without compromising transparency and flexibility. Furthermore, this combination harmonizes the material used for the top and bottom insulation layers, which are compatible with dry etching, thereby eliminating issues such as curling and delamination. The selection of all materials makes it less prone to failure when undergoing large angle bending to integrate with the GRIN lens. In applications where higher transparency is a priority, tensile strength requirements can be moderately relaxed. In such cases, Parylene-C, with better transparency, presents a suitable alternative as the insulation material.

**Table 1:** Comparison with graphene–ITO transparent electrodes

Reference	Graphene	ITO	Combined methods	Mechanical property	Feature size	Application
**This study**	Purchased from Xianfeng Nano Technology	Magnetron sputter	Direct transfer	Flexible	10 μm	Neural activity recording
Liang (2024)^43^	–	Magnetron sputter	Direct transfer	Rigid	20 μm	Optoelectronic synapses
Ahmed (2023)^44^	Synthesized in lab	Purchased from Sigma-Aldrich	Direct deposition	Rigid	–	Electrochemical detection
Fan (2023)^45^	–	Magnetron sputter	Direct transfer	Rigid	–	Light-emitting diodes
Lee (2021)^46^	Synthesized in lab	Atomic layer deposition	Direct deposition		–	Smart window
Silva (2020)^47^	Synthesized in lab	Magnetron sputter	Direct deposition	Rigid	–	–
Guo (2020)^48^	Synthesized in lab	Purchased from Zhuhai Kaivo	Direct transfer	Rigid	–	Electrochemical detection
Fioravanti (2020)^49^	Synthesized in lab	Purchased from Delta Technologies	Direct transfer	Rigid	–	Raman scattering
Yang (2019)^50^	Purchased from Xianfeng Nano Technology	Synthesized in lab	Direct deposition	Rigid	–	DNA biomolecule detection
Althagafi (2019)^51^	Synthesized in lab	Purchased from Sigma-Aldrich	Direct deposition	Rigid	–	Electrochemical detection
Yang (2017)^52^	Synthesized in lab	Magnetron sputter	Direct deposition	Rigid	0.5 mm	Photodetector
Hemasiri (2017)^53^	Synthesized in lab	Synthesized in lab	Physical Adsorption Particle Interlocking	–	–	–
Song (2016)^54^	Synthesized in lab	–	Operate separately in their respective roles	Flexible	–	Triboelectric generator
Liu (2016)^55^	Synthesized in lab	Purchased from Zhuhai Kaivo	Direct transfer	Flexible	–	–

Secondly, in electrode fabrication: Extensive research on TFNMEs has demonstrated their capability for multimodal neural activity recording without negative effects^21–24,27,28,30,39^, as detailed in Table 2. However, due to limitations in fabrication methods and material properties, most electrodes feature relatively large recording sites. A micro-etching technique and pre-treating the graphene with oxygen plasma were proposed to address the challenges in fabricating TFNMEs containing graphene. These advancements have successfully reduced electrode feature size to the single-cell level (recording site size of 20 μm and interconnects width of 10 μm), enabling the recording of single-neuron activity. The micro-etching technique employs multiple transfers of small-area graphene instead of a single transfer of large-area graphene, significantly reducing wrinkles and cracks during the transfer process and achieving high-quality large-area graphene transfers. Pre-treating the graphene with oxygen plasma specifically targets the process sequence where graphene is transferred before metal deposition. This approach addresses the poor step coverage of graphene and ensures low-impedance contacts between graphene and metals. The impedance of short-circuited metal interconnects to graphene is approximately 1 kΩ when graphene is transferred before metal deposition, compared to 30 kΩ when metal is deposited first. Notably, the electrochemical impedance of Gr–TFNMEs fabricated using both techniques is 1.8 MΩ at 1 kHz, which is lower than other Gr–TFNMEs^24^, demonstrating the effectiveness of these techniques for fabricating high-quality electrodes containing graphene.

**Table 2:** Comparison with other transparent flexible neural microelectrodes

Reference	Function materials	Insulation layer	Electrode size	Electrophysiology	Optophysiology	Animal status
**This study**	Graphene–ITO	Polyimide/Polyimide	314 μm^2^ (*d* = 20 μm)	Spikes & LFPs (intracortical)	Calcium imaging	Freely moving
Ledochowitsch^14^	ITO	Parylene C/Parylene C	7850 μm^2^ (*d* = 100 μm)	ECoG	Optogenetics	Head-fixed
Cho^22^	PEDOT:PSS-EG	PET-SU8	90000 μm^2^ (*w* = 300 μm)	LFPs & ECoG (cortical)	Optogenetics	Head-fixed
Dijk^23^	PEDOT:PSS	Parylene C/Parylene C	7850 μm^2^ (*d* = 100 μm)	LFPs (cortical)	Calcium imaging	Head-fixed
Zhang^24^	CNT	PDMS-SU8	10000 μm^2^ (*w* = 100 μm)	ECoG	Calcium imaging	Head-fixed
Thunemann^28^	Graphene	PET-SU8	10000 μm^2^ (*w* = 100 μm)	LFPs (cortical)	Calcium imaging & Optogenetics	Head-fixed
Ramezani^30^	id-DLG with PtNPs	Parylene C/Parylene C	314 μm^2^ (*d* = 20 μm)	Spikes (cortical)	Calcium imaging	Head-fixed

Thirdly, in the multimodal recording of intracortical neural activity: Previous research^21–24,27,28,39^ predominantly focused on application on the cortical surface, with fewer investigations on intracortical neural activity. Moreover, individual neuronal action potentials (also known as spikes) were rarely captured by TFNMEs due to the size of the electrodes. Although a recent study^30^ utilizing Gr–TFNMEs adjusted the recording site size to 20 μm through id-DLG and electroplating with PtNPs, successfully recording spikes, it was restricted to recording cortical neural activity in head-fixed mice due to imaging limitations. We integrated TFNMEs with the GRIN lens to successfully record neural activity in freely moving mice and captured intracortical spikes with TFNMEs for the first time. However, the recordings were limited to spontaneous neural activity, demonstrating the potential of Gr–ITO–TFNMEs as a versatile tool for multimodal neuroscience research. Future work should aim to expand electrode density to record a broader range of high spatiotemporal resolution neural activities and incorporate behavioral testing to capture nonlinear neural dynamics.

In conclusion, this study has proposed a new type of transparent flexible neural microelectrodes that can record intracortical multimodal neural activity in freely moving mice. As an effective tool, the electrodes pave the way for further advancements in understanding the complex dynamics of the brain with high spatiotemporal resolution for multifunctional neuroscience research.

## Materials and methods

### Fabrication processes of Gr–ITO–TFNMEs

(1) A 3 μm layer of PI was spin-coated at 3000 rpm and cured to form the bottom insulation layer after cleaning the 4-inch silicon wafer. (2) Alignment contours (~100 nm) were formed on the bottom PI by oxygen plasma treatment under a positive photoresist (MICROPOST S1805 G2) mask. It should be noted that the areas treated with oxygen plasma must be those intended for subsequent graphene transfer. These treated areas became hydrophilic, facilitating the transfer of graphene to the target areas and decreasing the possibility of forming excess bubbles that could damage the graphene. (3) Suspended self-help transfer few-layer graphene (Xianfeng Nano, 800–1000 Ω/▫) was aligned face-to-face with the alignment contours and sequentially transferred to the target areas after being released onto the surface of deionized water in a beaker. The graphene on PI was then baked at 150 °C for 15 min to dry residual moisture and partially melt polymethyl methacrylate (PMMA), enhancing contact between the graphene and the bottom PI, reducing wrinkle, and improving adhesion, as demonstrated in Fig. S1. The effect of wrinkles reduction was notably better after baking at 150 °C compared to 100 °C. After baking, the graphene was soaked in acetone for about 3 h to remove PMMA. (4) Metal interconnects were patterned through a lift-off process using AR-N4340 negative photoresist and thermally evaporated Cr/Au/Cr with thicknesses of 10/100/10 nm, respectively. Prior to thermal evaporation, the entire silicon wafer was treated with oxygen plasma to enhance the hydrophilicity of graphene. As shown in Fig. S2, this treatment facilitated the uniform and stable deposition of metals. Additionally, low-impedance contacts were formed between graphene and metals, validated by measuring the impedance of short-circuited metal interconnects to graphene, found to be approximately 1 kΩ. (5) The graphene was patterned by reactive ion etching (RIE) with AZ-6130 photoresist as the etch mask. (6) A 140 nm of ITO was magnetron sputtered to form the interface of recording sites with a lift-off process using a 99.99% purity indium tin oxide target material (In_2_O_3_:SnO_2_ = 90:10 wt%). The sputtering temperature was room temperature, the sputtering power was 150, the oxygen flux was 10 sccm, the argon flux was 70 sccm, the vacuum degree was 10^−7^ Torr, and the pressure was 8 mTorr. The thickness of the ITO functional layer was 140 nm. The sheet resistance of ITO was measured using a four-point probe method and found to be 228 Ω/▫. (7) A 3 μm layer of PI was spin-coated and cured as the top insulation layer. (8) The outline of Gr–ITO–TFNMEs, connection pads, and openings of recording sites were defined by RIE with AZ-4620 photoresist as the etch mask. (9) The entire silicon wafer was submerged in deionized water to release the electrodes.

### Electrochemical characterization

The CV and EIS were measured on a two-electrode system (CHI660E, Chenhua Inc., China) in 1× PBS solution (Cytiva HyClone). The experimental devices served as the working electrode, with a platinum electrode functioning as both the counter and reference electrode. CV characteristics were obtained by scanning from an initial voltage of −0.5 V to a final voltage of 0.5 V at a scan rate of 0.05 V/s and then scanning back to the initial voltage at the same rate. EIS was performed by applying a sinusoidal voltage with an amplitude of 0.05 V over a frequency range from 100 Hz to 100 kHz. An impedance of 1 kHz was used to represent the electrochemical impedance. An accelerated aging test was carried out to evaluate the capability for sustained recording by soaking the electrodes in PBS at 67 °C for 1 week (equivalent to eight weeks at 37 °C, with an acceleration factor of 8^40^). The impedance was measured every 24 h, comparing three different types of electrodes: Gr–ITO–TFNMEs, Gr–TFNMEs, and Au-based flexible microelectrodes (Au-FNMEs, as control).

### Fabrication process for the PDMS containing a low concentration of fluorescent microbeads

The microbeads were first thoroughly dispersed in the PDMS crosslinker at a 1:500 ratio with ultrasonic treatment for 30 minutes. This microbead-containing crosslinker was mixed with the PDMS prepolymer at a ratio of 1:10. After stirring and degassing to remove bubbles, the mixture was spin-coated onto pre-cured PDMS blocks at 2000 rpm. The excitation wavelength of the microbeads was 488 nm, and the emission wavelength was 520 nm.

### Animals

Male C57BL/6J mice were housed in a controlled environment with a 12-h light/dark cycle and ad libitum access to food and water. All animal experiments followed protocols approved by the IACUC (Institutional Animal Care and Use Committee) of Shenzhen Institute of Advanced Technology, Chinese Academy of Sciences (SIAT-IACUC-20221124-NS-NXXZX-ZDK-A2028-02).

### Surgery

Mice, approximately 8 weeks old, were anesthetized with isoflurane vapor (2% for induction and 1–1.5% for maintenance) mixed with O2 and fixed on a stereotaxic apparatus. Body temperature was maintained at 37.5 °C using a heating pad during surgery and anesthesia recovery. AAV2/9-hSyn-GCaMP7s virus (Shanghai Taitool Bioscience) was injected into the dorsal hippocampus (dHP) at AP: −2.1 mm, ML: +1.7 mm, DV: −1.4 mm from the surface of the skull with a 9° angle. A total of 400 nl of virus was injected at a rate of 15 nl/min. Then, the mouse’s scalp was sutured, and the mouse was placed on a heating pad to facilitate recovery. After that, the mouse was returned to its home cage.

Three weeks later, mice were remounted on a stereotaxic apparatus (Fig. S8a) for implantation. The scalp was carefully removed to expose the skull (Fig. S8b). Three holes were made in the skull with a cranial drill, and screws were inserted. The screw near the bregma was used as the reference electrode (red arrow in Fig. S8c), while the screw near the lambda was used as the ground electrode (black arrow in Fig. S8c).

A 1.2 mm-diameter hole (blue arrow in Fig. S8c) was drilled above the hippocampus to expose the dura mater. The dura mater was then removed with a needle tip, exposing the cortical surface, which was washed with ACSF (Fig. S8d). The cortex was aspirated using a 0.4 mm diameter needle connected to a vacuum pump (Fig. S8e, f), slowly aspirating the tissue until the white-colored corpus callosum was exposed (Fig. S8g). Once the white-colored corpus callosum was aspirated, the hippocampal surface was fully exposed (Fig. S8h). The protective cap was held with a clamp, and the surface of the relay lens integrated with Gr–ITO–TFNMEs was gently pressed against the hippocampus, using the edge of the cranial hole as the reference point, to a depth of 1.15 mm (Fig. S8i, j). Dental cement was then used to encase the exposed skull screws, baseplate, objective lens, and other components, forming a solid and stable structure (Fig. S8k). Finally, the ground and reference electrodes were soldered in place (Fig. S8l), and the exposed wires were covered with dental cement (Fig. S8m). After 1 week of recovery and adaptation, electrophysiology recording and calcium imaging were simultaneously conducted in freely moving mice.

### Electrophysiology recording and calcium imaging

The signals were sampled at 30 kHz and transmitted to the data acquisition equipment (NeuroStudio System, Jiangsu Brain Medical Technology Co.). The electrical cables were connected to slip rings to avoid entanglement with the TINIscope system. Additionally, a shielding mesh (100 cm × 80 cm × 120 cm) covered the system to minimize interference with electrophysiological signals. Calcium imaging was acquired at 12.5 frames per second by the TINIscope system. Electrophysiological signals and calcium imaging were synchronized by sending TTL pulses to Neurostudio and controlling the LED signal lights within the behavioral field of view.

### Histology

After recording, mice were sacrificed and transcranial perfused with saline followed by 4% paraformaldehyde (Sigma) in PBS. The brains were kept in 4% paraformaldehyde at 4 °C for 24 h and subsequently immersed in 30% sucrose for 72 h before being sliced into 40-μm coronal sections and imaged under a fluorescence microscope (Olympus MVX10).

### Imaging data and electrophysiology analysis and correlation

Calcium imaging was processed with motion correction^41^ and constrained non-negative matrix factorization^42^, which facilitated identifying individual neurons and the acquisition of neuron traces. Electrophysiological signals were analyzed and processed utilizing the Offline Sorter and NeuroExplorer software.

To analyze the electrophysiological signal (LFP) and calcium signal correlation, we first identified the peaks in the normalized calcium signal (average calcium intensity). Next, we calculated the time-averaged LFP power spectral density around the onset times of these peaks. Pearson correlation coefficients were computed for each recording site to quantify the relationship between the calcium peaks and the average LFP power spectral density. The same procedure was applied to calculate the correlation between the calcium trace and the spike raster.

## Supplementary information

Supplementary video

Supplementary information
