# Supplementary material for: Transparent, flexible graphene–ITO-based neural microelectrodes for simultaneous electrophysiology recording and calcium imaging of intracortical neural activity in freely moving mice
Source: Microsyst Nanoeng. 2025 Feb 25;11:32. doi: 10.1038/s41378-025-00873-y (PMC11850855; doi:10.1038/s41378-025-00873-y)
Supplement: Supplementary file 2 — Supplementary information [file 41378_2025_873_MOESM2_ESM.docx]

**Supplementary Information**

**Transparent, flexible graphene-ITO-based neural microelectrodes for simultaneous electrophysiology recording and calcium imaging of intracortical neural activity in freely moving mice**

Miao Yuan^1,2,†^, Fei Li^3,4,†^, Feng Xue^5^, Yang Wang^1,2^, Baoqiang Li^3^, Rongyu Tang^1,2^, Yijun Wang^1,2,6^, Guo-Qiang Bi^3,4,5, *^, Weihua Pei^1,2,6*^

^1^Laboratory of Solid-State Optoelectronics Information Technology, Institute of Semiconductors, Chinese Academy of Sciences, Beijing 100083, China.

^2^Institute of Semiconductors, University of Chinese Academy of Sciences, Beijing 10049, China.

^3^Interdisciplinary Center for Brain Information, the Brain Cognition and Brain Disease Institute, Shenzhen Institutes of Advanced Technology, Chinese Academy of Sciences, Shenzhen 518055, China.

^4^Shenzhen-Hong Kong Institute of Brain Science, Shenzhen 518055, China.

^5^Hefei National Research Center for Physical Sciences at the Microscale, University of Science and Technology of China, Hefei 230026, China.

^6^School of Future Technology, University of Chinese Academy of Sciences, Beijing 100049, China

*Corresponding authors. Guo-Qiang Bi (gqbi@ustc.edu.cn), Weihua Pei (peiwh@semi.ac.cn)

†Equally contributed to this work.

This PDF file includes:

Supplementary Note S1-S2

Supplementary Table S1

Supplementary Fig. S1-S13

Supplementary Note S1. Fabrication processes of Gr-TFNMEs.

(1) A 5 μm layer of PI was spin-coated at 2000 rpm and cured to form the bottom insulation layer after cleaning the 4-inch silicon wafer.

(2) Alignment contours (~100 nm) were formed on the bottom PI by oxygen plasma treatment under a positive photoresist (MICROPOST S1805 G2) mask. It should be noted that the areas treated with oxygen plasma must be those intended for subsequent graphene transfer. These treated areas became hydrophilic, facilitating the transfer of graphene to the target areas without the formation of excess bubbles that could damage the graphene.

(3) Suspended self-help transfer few-layer graphene (Xianfeng Nano, 800~1000 Ω/□) was aligned face-to-face with the alignment contours and sequentially transferred to the target areas after being released onto the surface of deionized water in a beaker. The graphene on PI was then baked at 150℃ for 15 minutes to dry residual moisture and partially melt polymethyl methacrylate (PMMA), enhancing contact between the graphene and the bottom PI, reducing wrinkles, and improving adhesion, as demonstrated in Fig. S1. The effect of wrinkles reduction was notably better after baking at 150°C compared to 100°C. After baking, the graphene was soaked in acetone for about 3 hours to remove PMMA.

(4) Metal interconnects were patterned through a lift-off process using AR-N4340 negative photoresist and thermally evaporated Cr/Au/Cr with thickness of 10/100/10 nm, respectively. Prior to thermal evaporation, the entire silicon wafer was treated by oxygen plasma to enhance the hydrophilicity of graphene. As shown in Fig. S2, this treatment facilitated the uniform and stable deposition of metals. Additionally, low-impedance contacts were formed between graphene and metals, validated by measuring the impedance of short-circuited metal interconnects to graphene, found to be approximately 1 kΩ.

(5) The graphene was patterned by reactive ion etching (RIE) with AZ-6130 photoresist as the etch mask.

(6) An 1μm-thick SU-8 2002 (MicroChem Corp.) insulation layer with outline of Gr-TFNMEs, connection pads and openings of recording sites were patterned with photolithography, followed by 10 s of isopropanol/deionized water rinse to cleanse SU-8 residue.

(7) The outline of bottom PI for Gr-TFNMEs, connection pads and openings of recording sites were defined by RIE with AZ-4620 photoresist as the etch mask.

(8) The entire silicon wafer was submerged in deionized water to release the electrodes.

Supplementary Note S2. Fabrication processes of ITO-TFNMEs.

(1) A 3 μm layer of PI was spin-coated at 3000 rpm and cured to form the bottom insulation layer after cleaning the 4-inch silicon wafer.

(2) A 140 nm of ITO was sputtered to form the interconnects and interface of recording sites through a lift-off process using AR-N4340 negative photoresist.

(3) A 3 μm layer of PI was spin-coated and cured as the top insulation layer.

(4) The outline of ITO-TFNMEs, connection pads and openings of recording sites were defined by RIE with AZ-4620 photoresist as the etch mask.

(5) The entire silicon wafer was submerged in deionized water to release the electrodes.

Supplementary Table S1. Comparison with electrode insulation materials

| **Insulation material** | **Young modulus** | **Tensile strength** | **Patterning methods** | **Transparency** |
| --- | --- | --- | --- | --- |
| **Polyimide (PI 2611)** | 8.5 GPa | 350 MPa | Photolithography and etching | ~80% |
| Parylene-C | 2.76 GPa | 69 MPa | Photolithography and etching | ~98% |
| SU-8 | 2.0 GPa | 60 MPa | Photolithography | ~95% |
| PDMS | ~0.1-3 MPa | 6.7 MPa | Photolithography and molding | ~95% |


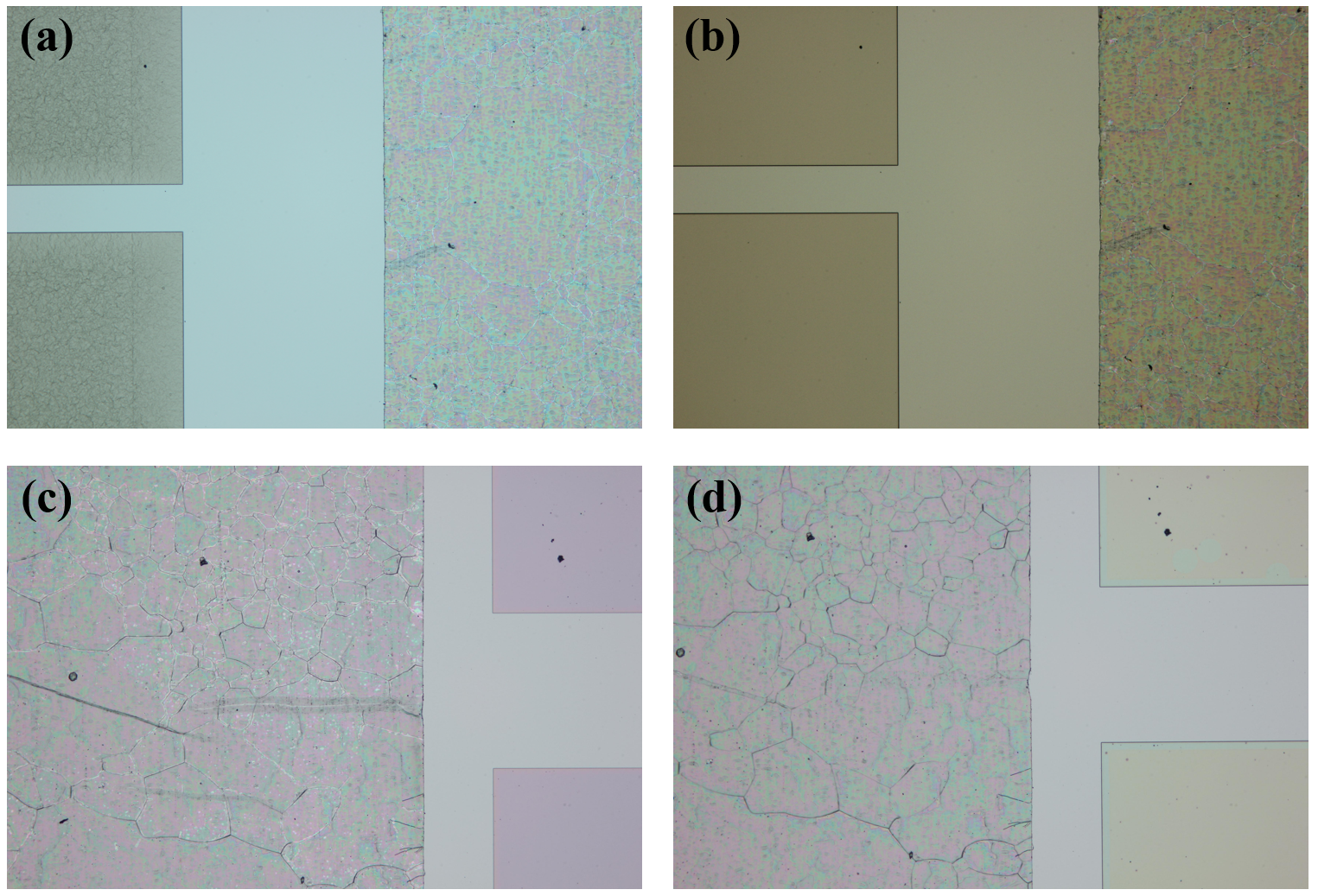


Supplementary Fig. S1. The effects of baking temperature for graphene wrinkles. (a) Before baking at 100 ℃. (b) After baking at 100 ℃. (c) Before baking at 150 ℃. (d) After baking at 150 ℃.


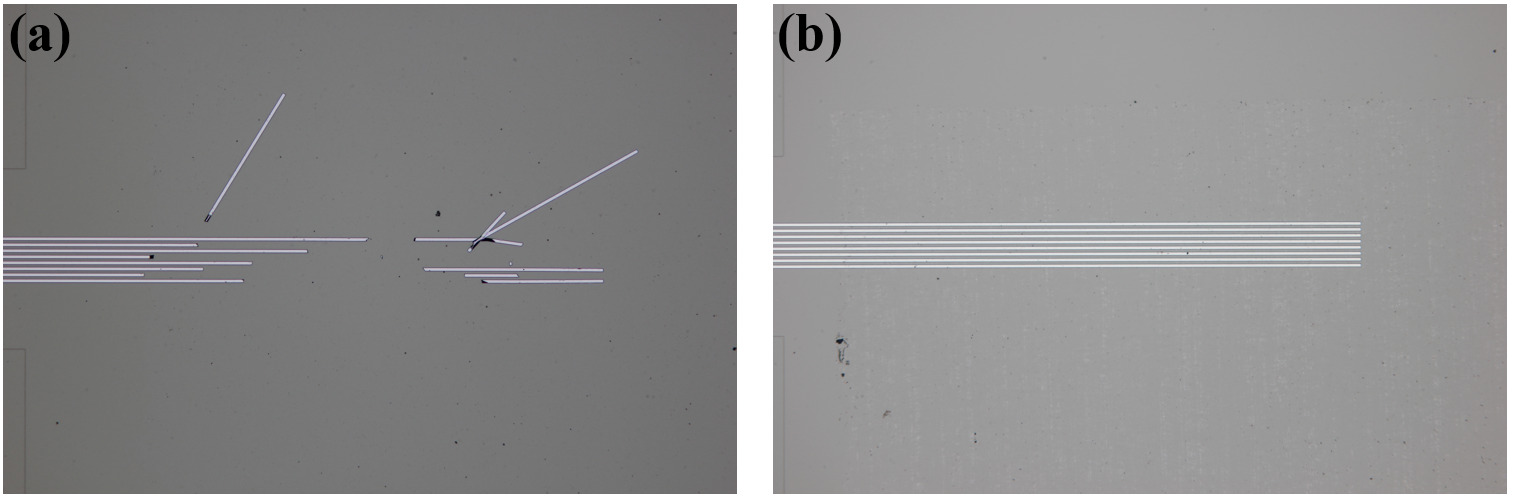


Supplementary Fig. S2. The effects of pre-oxygen plasma treatment. (a) Without pre-oxygen plasma treatment. (b) With pre-oxygen plasma treatment.


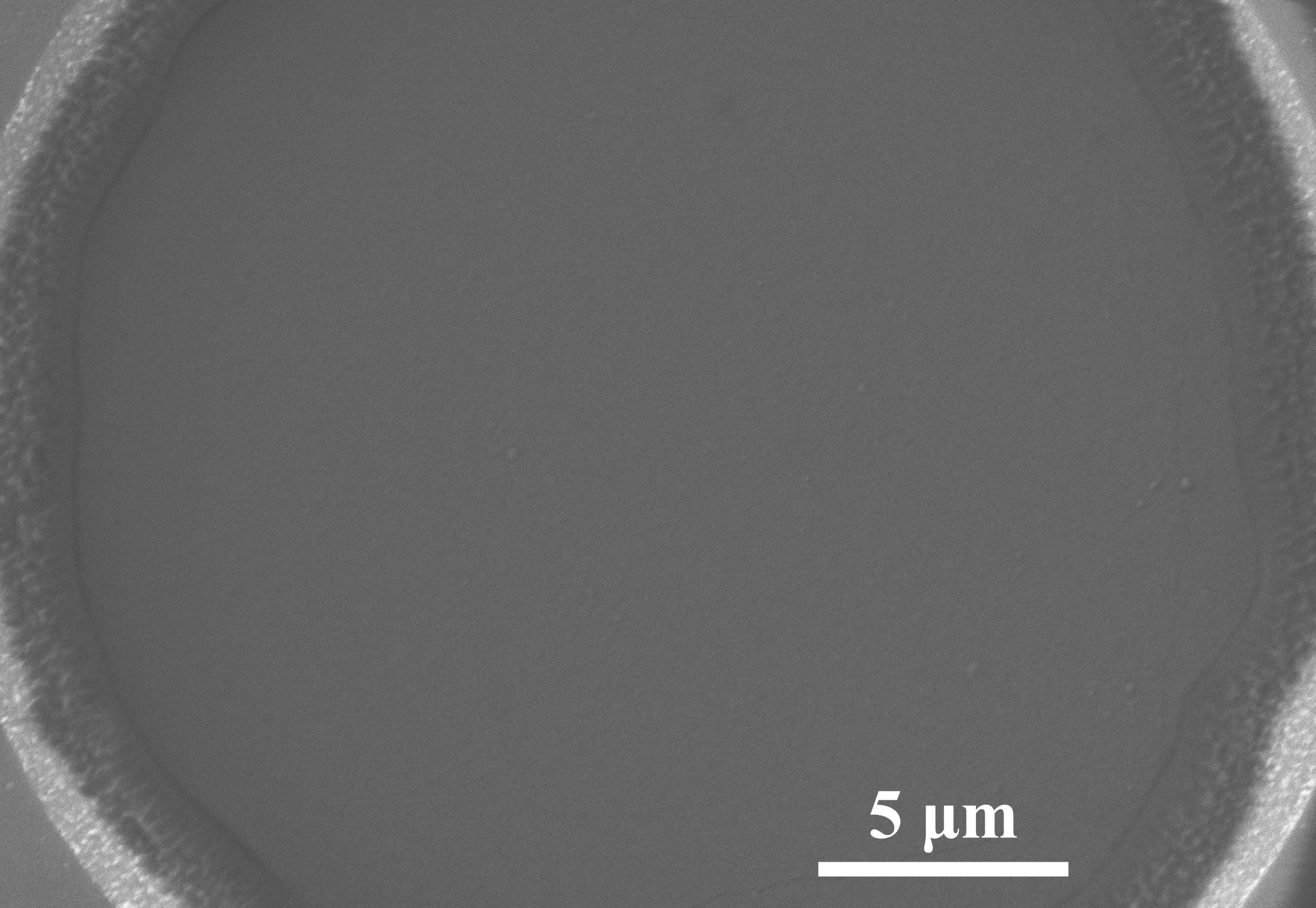


Supplementary Fig. S3. The SEM of recording site.


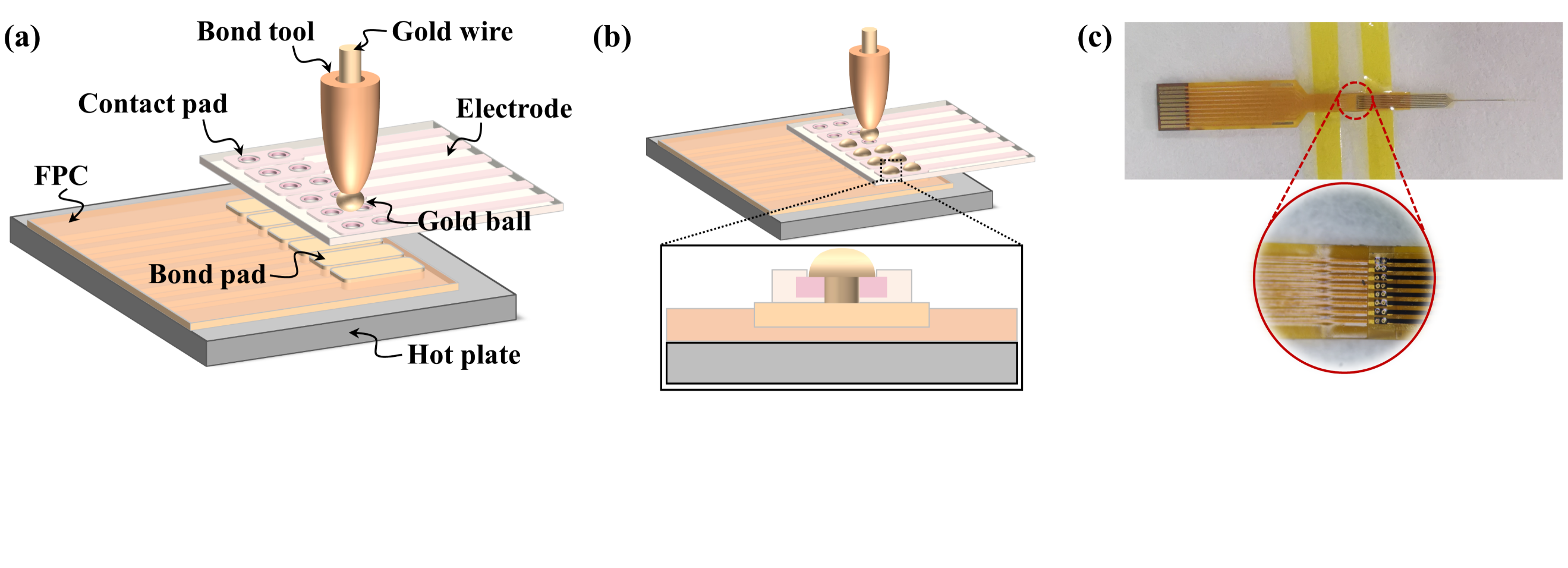


Supplementary Fig. S4. The illustration of Gr-ITO-TFNMEs connecting FPC by gold ball bonding. (a) The schematic diagram of Gr-ITO-TFNMEs connecting FPC by gold ball bonding. (b) The cross-section of Gr-ITO-TFNMEs connecting FPC by gold ball bonding. (c) Actual photographs of Gr-ITO-TFNMEs connecting FPC by gold ball bonding.


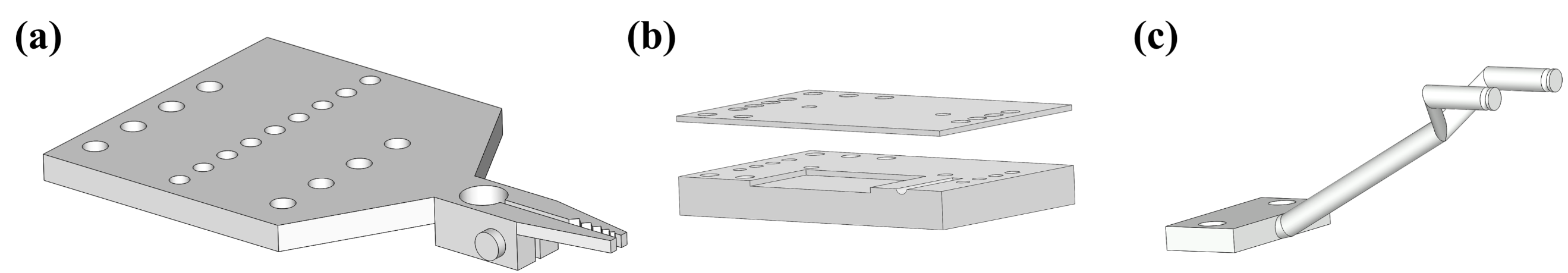


Supplementary Fig. S5. Design integration tools. (a) Lens holder. (b) Slide holder. (c)Wire tool.

Supplementary Fig. S6. The electrochemical impedance at 1kHz for six recording sites of ITO-TFNMEs.


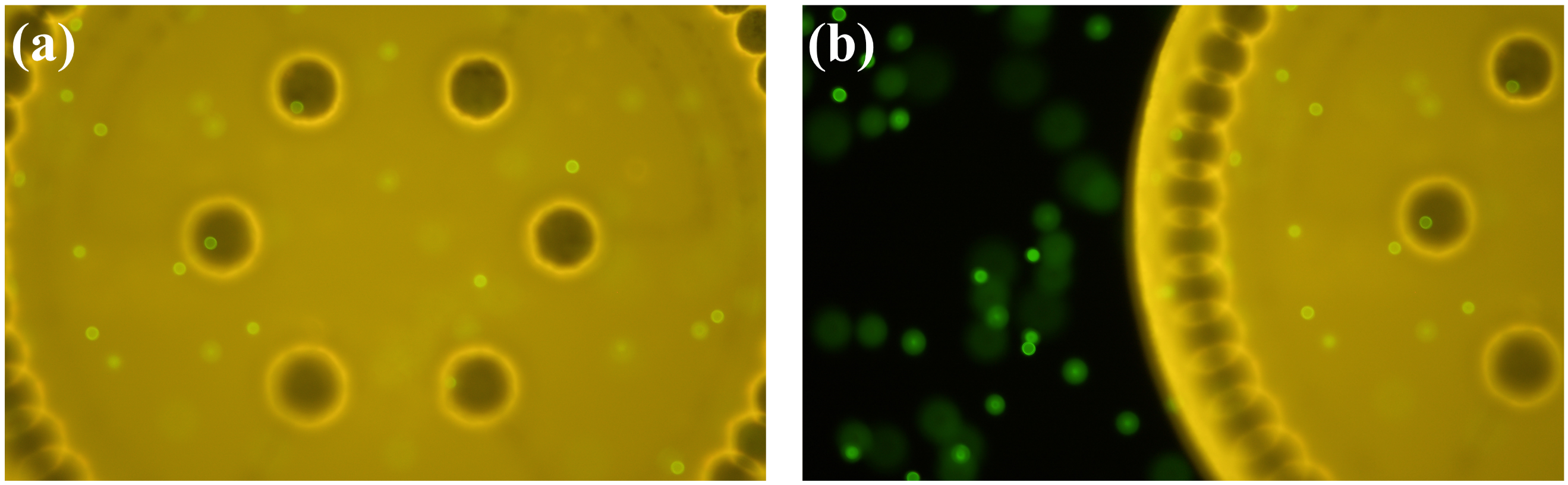


Supplementary Fig. S7. Higher magnification fluorescent polystyrene microbeads imaging of Gr-ITO-TFNMEs. (a) The fluorescent microbeads imaging under recording sites and interconnects. (b) The fluorescent microbeads imaging under both FOV and out of FOV of Gr-ITO-TFNMEs.


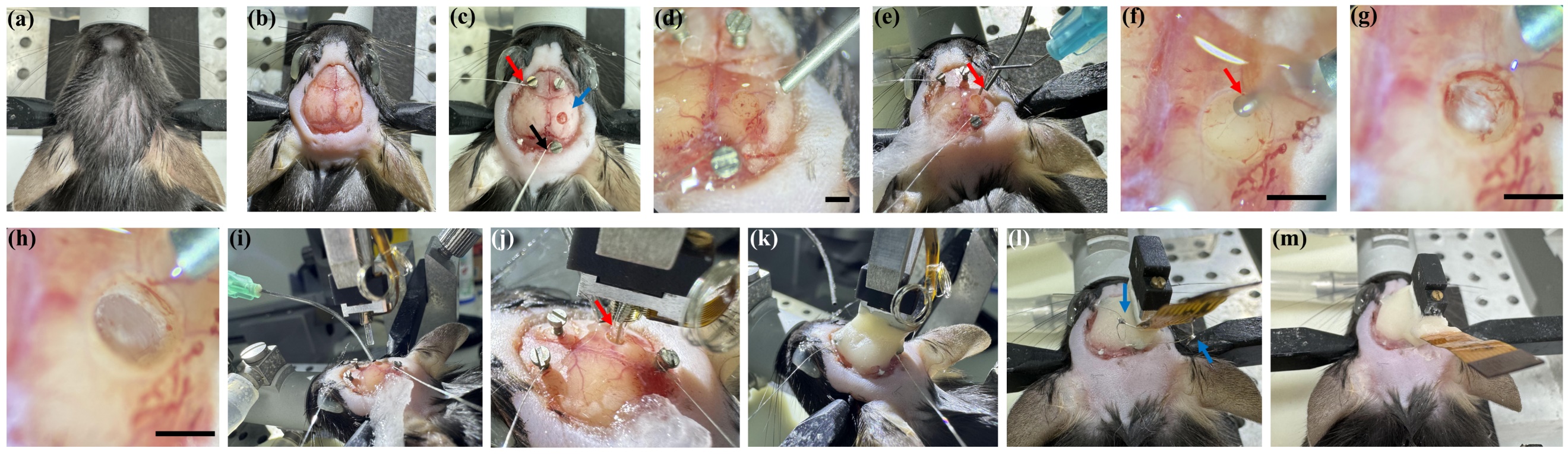


Supplementary Fig. S8. Detailed operation processes. (a) The mouse is fixed onto the stereotaxic apparatus. (b) The scalp is removed to expose the skull. (c) Three holes are drilled into the skull using a cranial drill, and three screws are inserted. The screw near the bregma point is used as the reference electrode (red arrow), and the screw near the lambda is used as the ground electrode (black arrow). A 1.2 mm diameter circular hole (blue arrow) is drilled above the hippocampus to expose the dura mater. (d) The dura mater is removed with a needle tip, exposing the cortex, which is then washed with ACSF. (e-f) A 0.4 mm diameter needle (red arrow) connected to a vacuum pump is used to aspirate the cortex. (g) The cortex is slowly aspirated until the white-colored corpus callosum is exposed. (h) After aspirating the white-colored corpus callosum, the hippocampal surface is exposed. (i-j) The protective cap is held with a clamp, and the surface of the relay lens (red arrow) is gently pressed against the hippocampus, using the edge of the cranial hole as the reference point, to a depth of 1.15 mm. (k) Dental cement is used to encase the exposed skull screws, baseplate, objective lens, and other components, forming a solid structure. (l) The ground and reference electrodes are soldered in place (blue arrow). (m) The exposed wires are covered with dental cement. Scale bar: 1 mm for (d), (f), (g), (h).


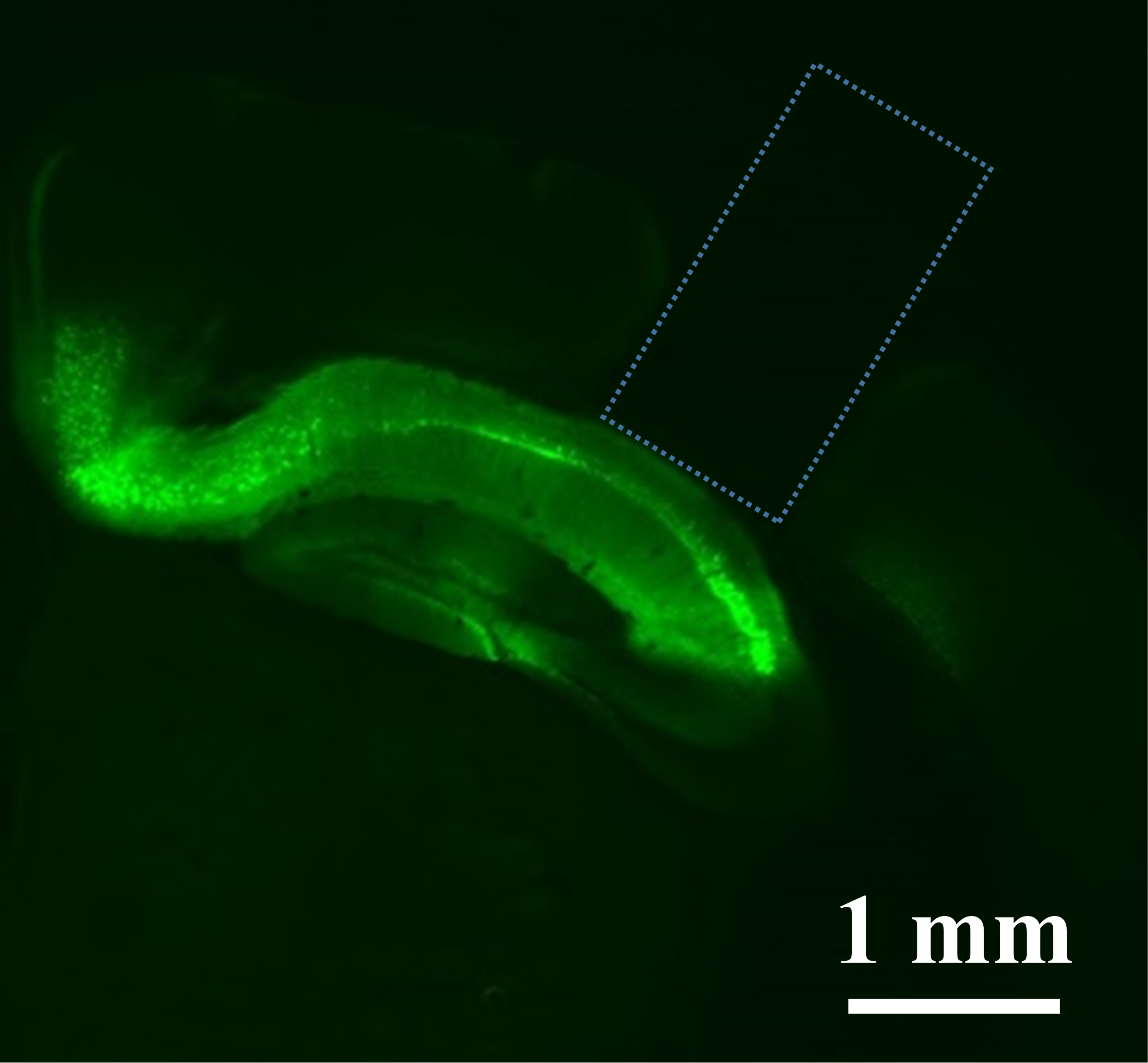


Supplementary Fig. S9. An image of CA1 region with GRIN lens implant.


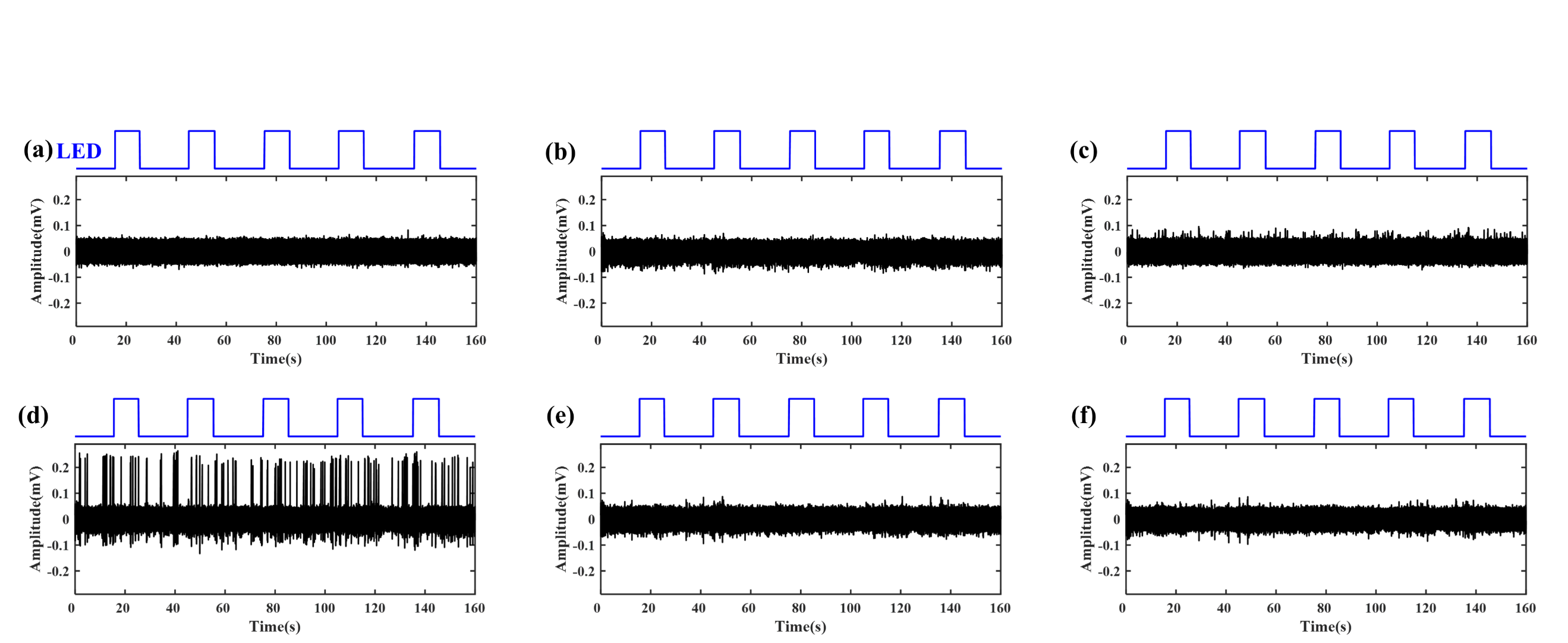


Supplementary Fig. S10. The effect of the excitation light over six recording sites. (The blue line represents the on-off state of the blue LED excitation light. The plot within the frame shows the changes in the 300 Hz high-pass filtered signal during synchronized times, where the baseline noise indicates the impact of light-induced artifacts.) (a) Recording site 1. (b) Recording site 2. (c) Recording site 3. (d) Recording site 4, with action potentials occurrence. (e) Recording site 5. (f) Recording site 6.


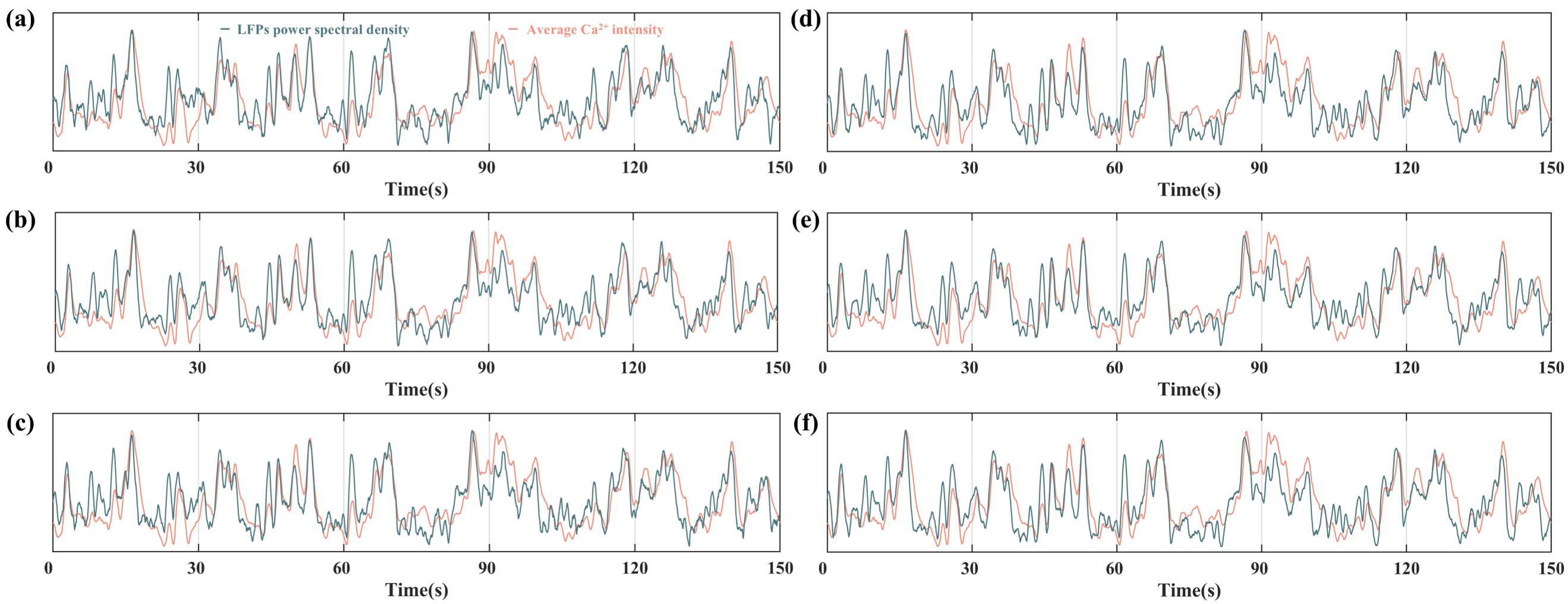


Fig. S11 The recalibrated plot of the average calcium intensity and the LFP power spectral density after shifting the average calcium intensity forward by 1.8 s across six recording sites. (a) Recording site 1. (b) Recording site 2. (c) Recording site 3. (d) Recording site 3. (e) Recording site 5. (f) Recording site 6.


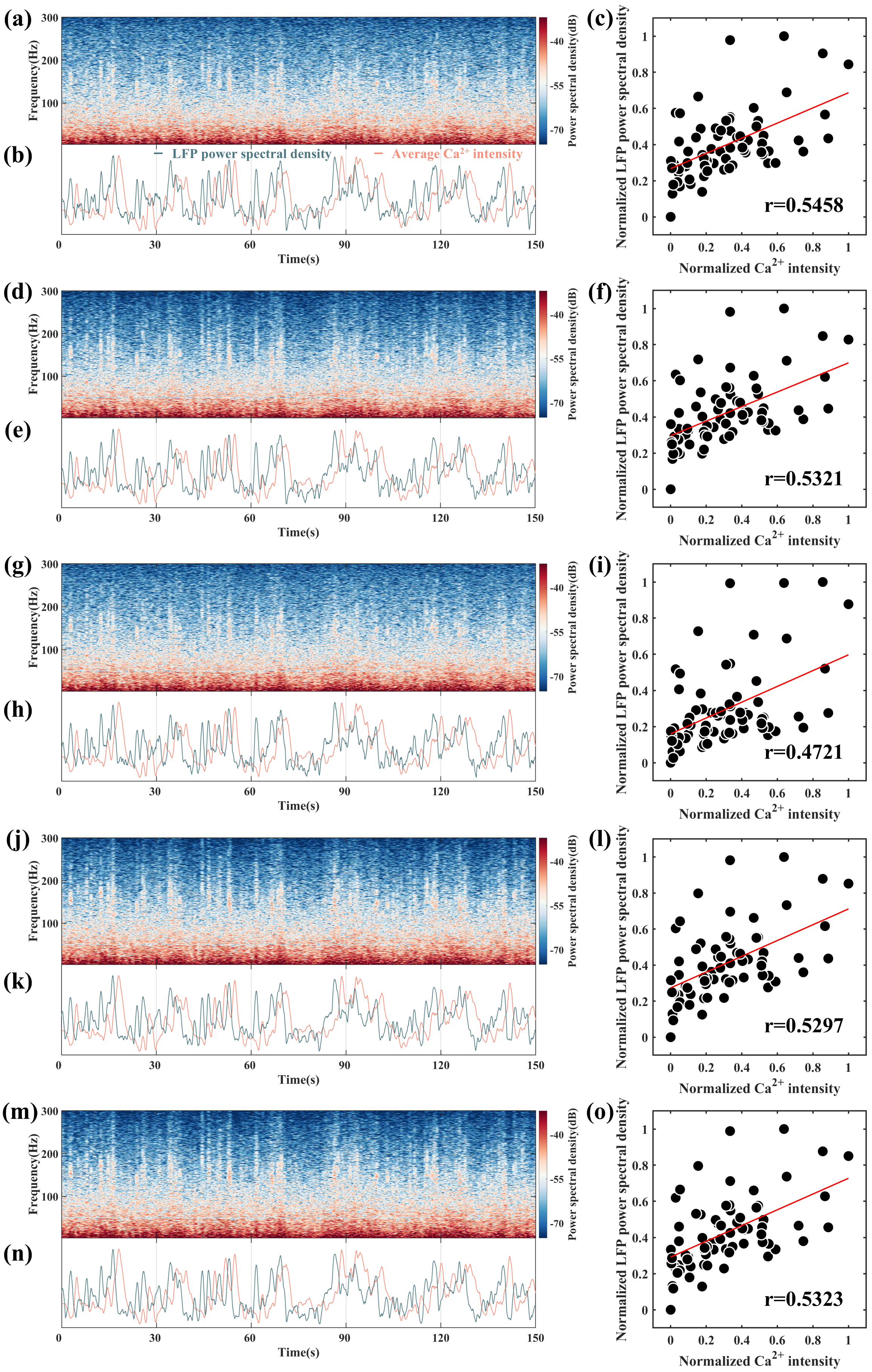


Supplementary Fig. S12. The power spectral densities of the LFPs from the other five recording sites in Fig. 6(d) and their correlations with the average calcium intensity. (a-c) Recording site 1. (d-e) Recording site 2. (g-h) Recording site 3. (j-k) Recording site 5. (m-n) Recording site 6.


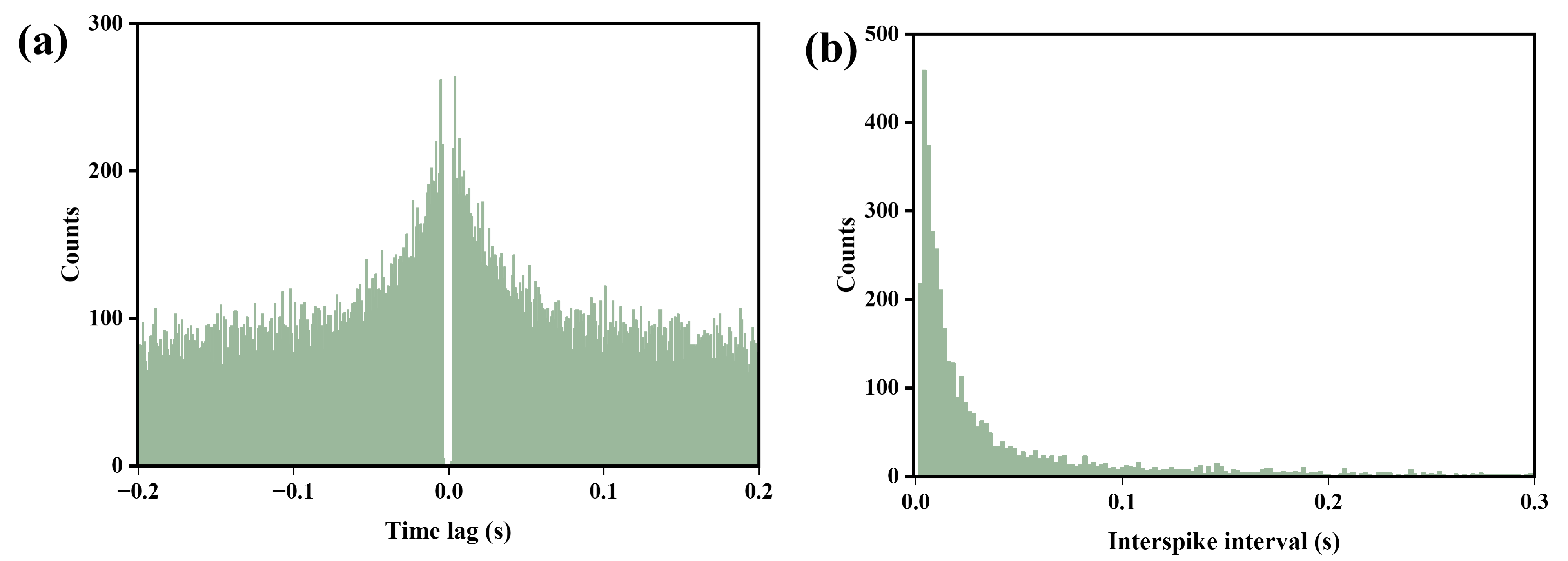


Supplementary Fig. S13. (a) The auto-correlogram of the detected neuron. (b) the inter-spike interval histogram of the detected neuron.
